# Supplementary material for: The effect of bacteria on planula-larvae settlement and metamorphosis in the octocoral Rhytisma fulvum fulvum
Source: PLoS One. 2019 Sep 30;14(9):e0223214. doi: 10.1371/journal.pone.0223214 (PMC6768449; doi:10.1371/journal.pone.0223214)
Supplement: S1 Fig — (DOCX) [file pone.0223214.s001.docx]

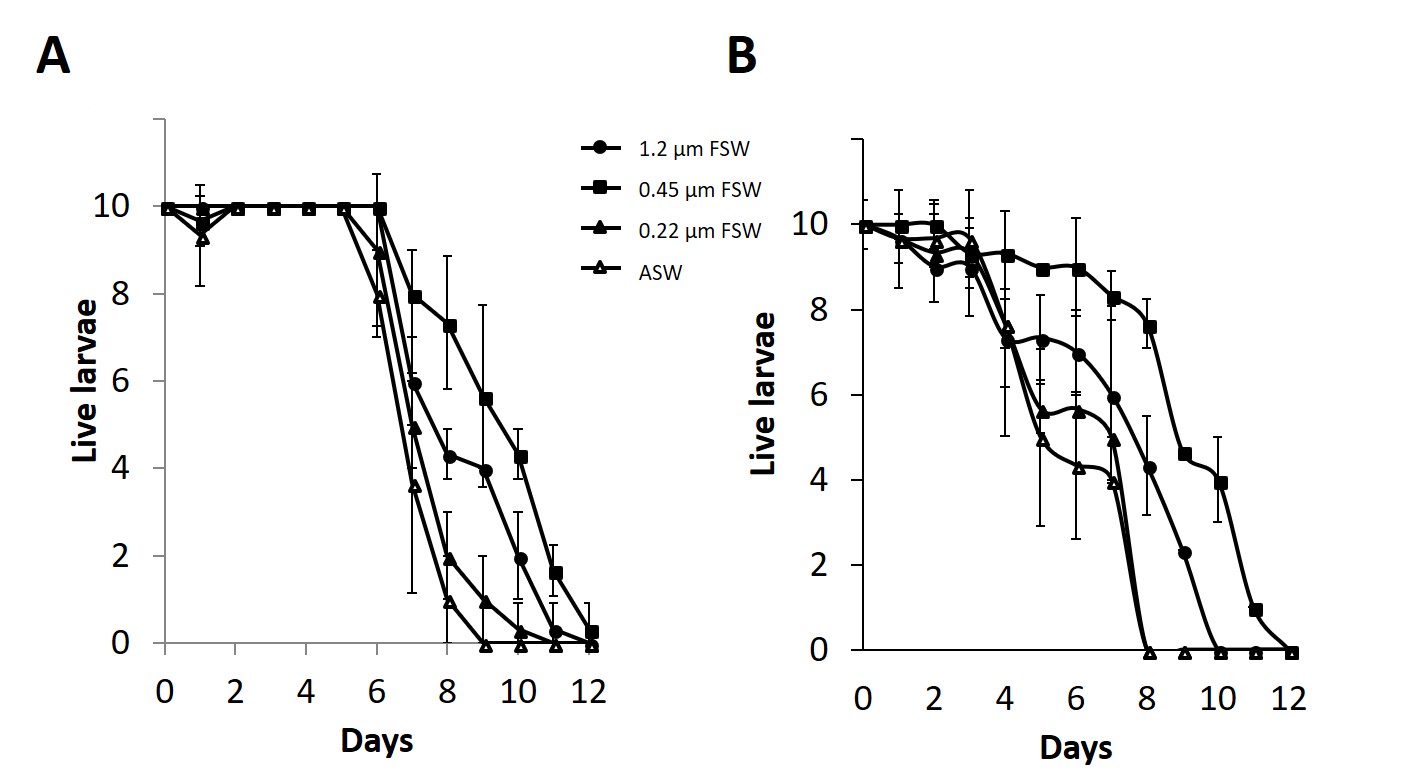


**S1 Fig. Survival of planulae the octocoral *Rhytisma fulvum fulvum* maintained with different filtered seawater (FSW).** Water was filtered through 1.2 µm, 0.45 µm, 0.22 µm, and autoclaved sea water (ASW) A. Darkness. B. Photoperiod, light:dark (12:12h). Water was not exchanged during the culture period. Data are means ± S.D. (n=3, 10 planulae per replicate).
