# Supplementary material for: The effect of bacteria on planula-larvae settlement and metamorphosis in the octocoral Rhytisma fulvum fulvum
Source: PLoS One. 2019 Sep 30;14(9):e0223214. doi: 10.1371/journal.pone.0223214 (PMC6768449; doi:10.1371/journal.pone.0223214)
Supplement: S2 Fig — (DOCX) [file pone.0223214.s002.docx]

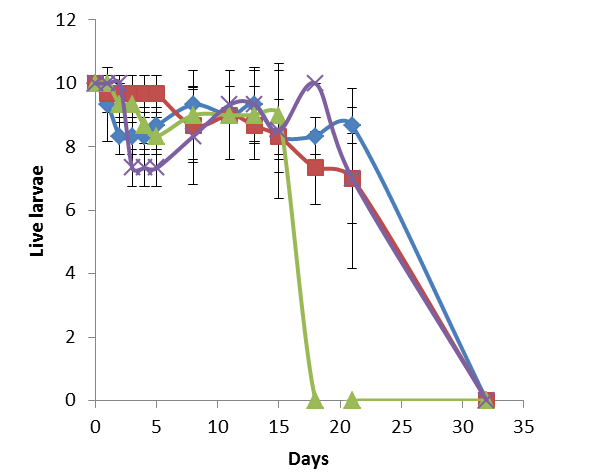

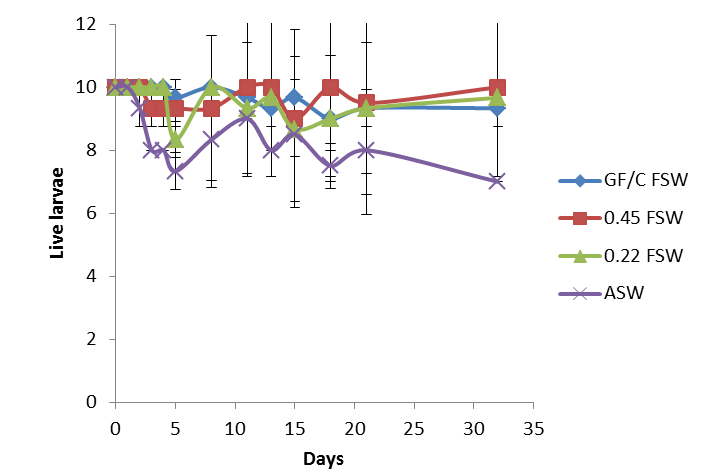


Water exchange

No water exchange

**PLASTIC**

**GLASS**

Water exchange

No water exchange


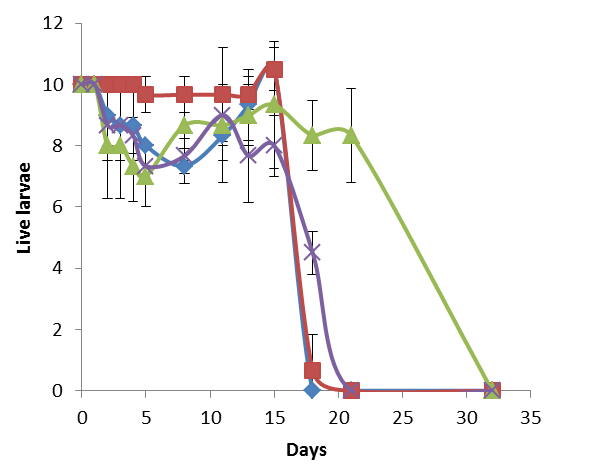

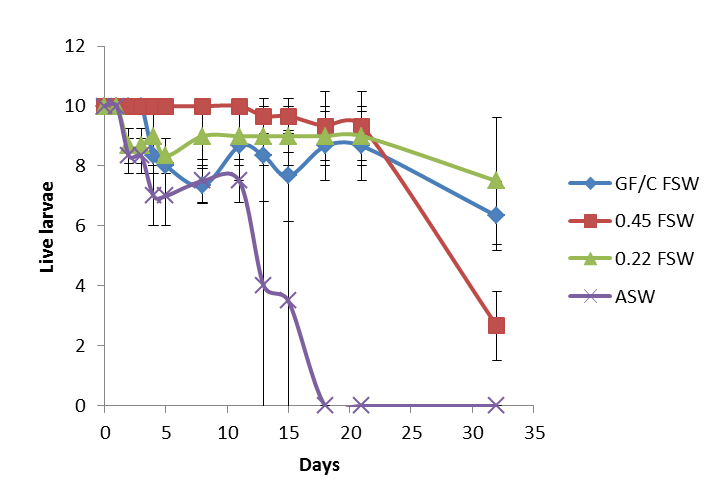


**S2 Fig. Survival of planulae the octocoral *Rhytisma fulvum fulvum* maintained in plastic and glass plates and different filtered sea water (FSW): 1.2 µm, 0.45 µm, 0.22 µm, and autoclaved sea water (ASW).** Larvae were harvested in 2015. Cultures were maintained under (12:12h) with our without water exchange every other day. Data are means ± S.D. (n=3, 10 planulae per replicate).
