# Supplementary material for: The effect of bacteria on planula-larvae settlement and metamorphosis in the octocoral Rhytisma fulvum fulvum
Source: PLoS One. 2019 Sep 30;14(9):e0223214. doi: 10.1371/journal.pone.0223214 (PMC6768449; doi:10.1371/journal.pone.0223214)
Supplement: S3 Fig — (DOCX) [file pone.0223214.s003.docx]

**A**

ASW

0.22 µm FSW

0.45 µm FSW

1.2 µm FSW


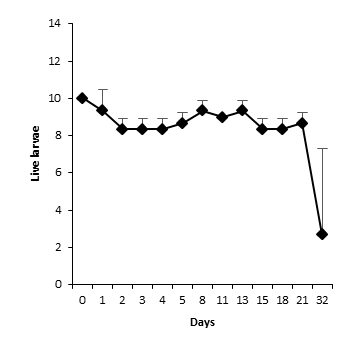

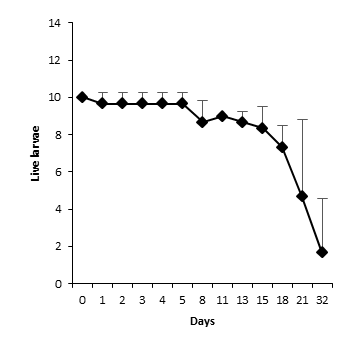

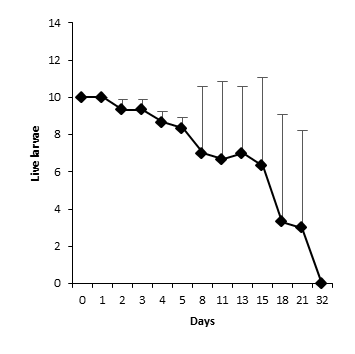

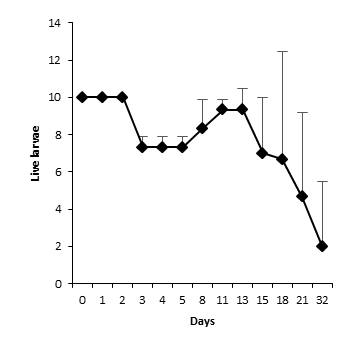


**S3 Fig. Survival of planulae the octocoral *Rhytisma fulvum fulvum* maintained without water exchange with different filtered sea water (FSW): 1.2 µm, 0.45 µm, 0.22 µm, and autoclaved sea water (ASW).** Larvae were harvested in 2015. This experiment was carried out in parallel with the cultures maintained with water exchange shown in Fig. 2. Cultures were maintained under light:dark cycles (12:12h). Data are means ± S.D. (n=3, 10 planulae per replicate).
