# Supplementary material for: The effect of bacteria on planula-larvae settlement and metamorphosis in the octocoral Rhytisma fulvum fulvum
Source: PLoS One. 2019 Sep 30;14(9):e0223214. doi: 10.1371/journal.pone.0223214 (PMC6768449; doi:10.1371/journal.pone.0223214)
Supplement: S4 Fig — (DOCX) [file pone.0223214.s004.docx]

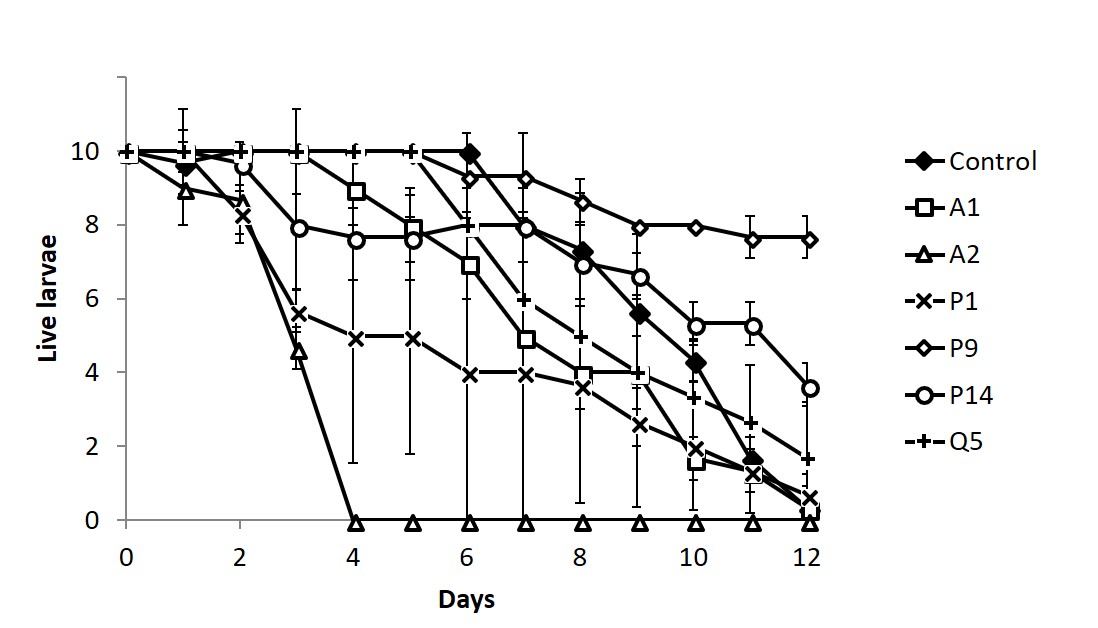


**S4 Fig. Survival of the octocoral *Rhytisma fulvum fulvum* larvae with different bacteria in cultures without water exchange.** Cultures were maintained without water change in total darkness (n=3, 10 planulae per replicate). The experiment was carried out with planulae of the 2014 batch in 0.45 µm FSW and bacteria were added at the beginning of the experiment at a concentration of 10^3^ CFU mL^-1^. Six bacteria isolates, comprising two *Alteromonas macleodii* strains (P9 and and P14), *Mameliella atlantica* (strain Q5), *Marinobacter litoralis* (Strain A1), *Thalassospira profundimaris* (Strain P1) and the actinobacterium *Kokuria rosea* (strain A2) were added at the beginning of the experiment. Whereas the planulae maintained in the control cultures did not survive more than 12 days, the addition of the two *Alteromonas* *macleodii* strains resulted in a statistically significant increase in the survival of the planulae on day 12 (Figure 2S, Mann-Whitney test p=0.1). Despite this high survival rate, no signs of metamorphosis were observed in any of the cultures after 12 days.
