# Supplementary material for: The effect of bacteria on planula-larvae settlement and metamorphosis in the octocoral Rhytisma fulvum fulvum
Source: PLoS One. 2019 Sep 30;14(9):e0223214. doi: 10.1371/journal.pone.0223214 (PMC6768449; doi:10.1371/journal.pone.0223214)
Supplement: S5 Fig — (DOCX) [file pone.0223214.s005.docx]

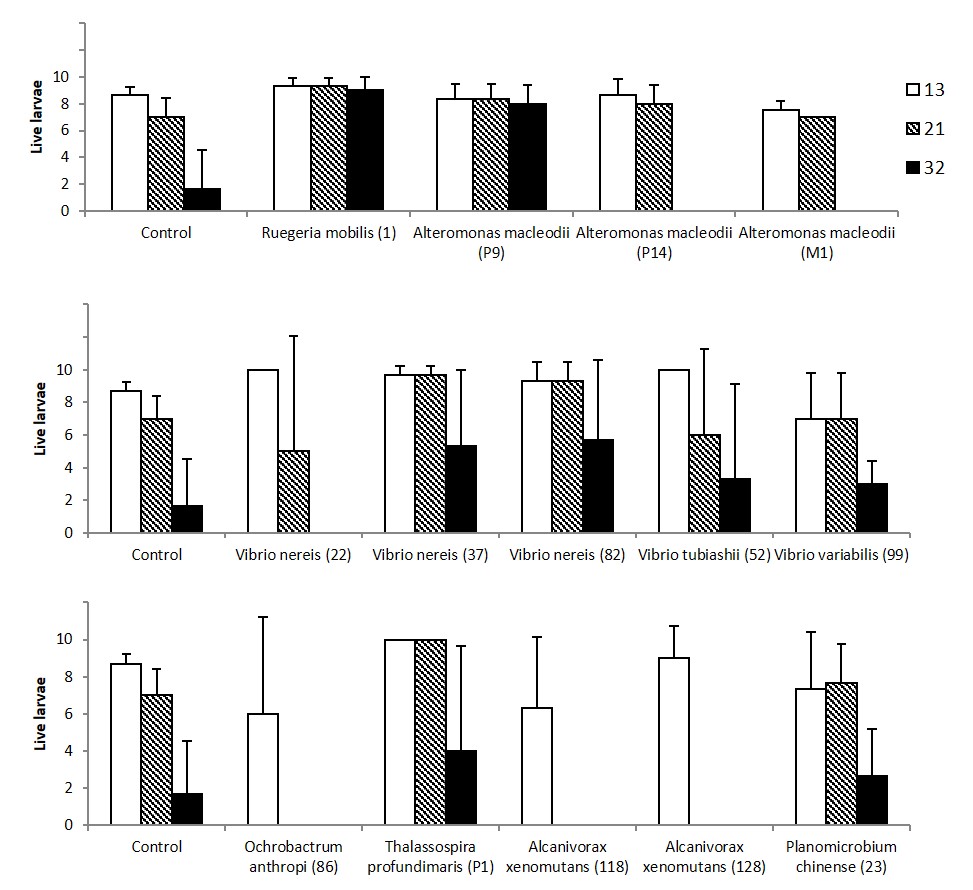


**S5 Fig.** **Survival rates of *Rhytisma fulvum fulvum* planulae in the presence of different native bacteria in cultures without water exchange.** The experiment was carried out with planulae of the 2015 batch maintained in 0.45 µm FSW without water exchange and bacteria were added at the beginning of the experiment at a final concentration of 10^3^ CFU mL^-1^. The cultures were maintained under a light:dark cycle (12:12h). Survival was measured on days 13, 21 and 32 of incubation. Data are presented as means ± S.D., (n=3, 10 individuals per replicate). The experiment confirmed the beneficial effect of isolate P9 (*Alteromonas macleodii*) observed in the 2014 experiment in cultures without water change (Figure 2S), yielding a survival rate of 90 % ± 10 on day 32 in comparison to 16.7 % ± 28.9 obtained in the control. In contrast, another two strains of the same species (Strains P14 and M1) were detrimental to planulae survival under the test conditions. A Roseobacter-clade member, *Ruegeria mobilis*, also promoted survival in comparison to the control achieving an 80± 14.1% on day 32. High variability was found among the *Vibrio* strains tested, with two of them (isolates 37 and 82) promoting a survival rates of 53.3% ± 46.2 and 56.7% ± 49.3, respectively but no survival was observed for strain 22 on day 32. The two *Alcalinovorax xenomutans* isolates (strains 118 and 128) and *Ochrovactrum anthropi* (strain 86) did not allow survival of the planulae beyond day 13. Despite the high survival obtained with the addition of some of the strains in comparison to the control in the cultures maintained without water exchange, the planulae did not undergo full metamorphosis under these conditions, with only very loosely attached planulae being observed.
