# Supplementary material for: The effect of bacteria on planula-larvae settlement and metamorphosis in the octocoral Rhytisma fulvum fulvum
Source: PLoS One. 2019 Sep 30;14(9):e0223214. doi: 10.1371/journal.pone.0223214 (PMC6768449; doi:10.1371/journal.pone.0223214)
Supplement: S6 Fig — (DOCX) [file pone.0223214.s006.docx]

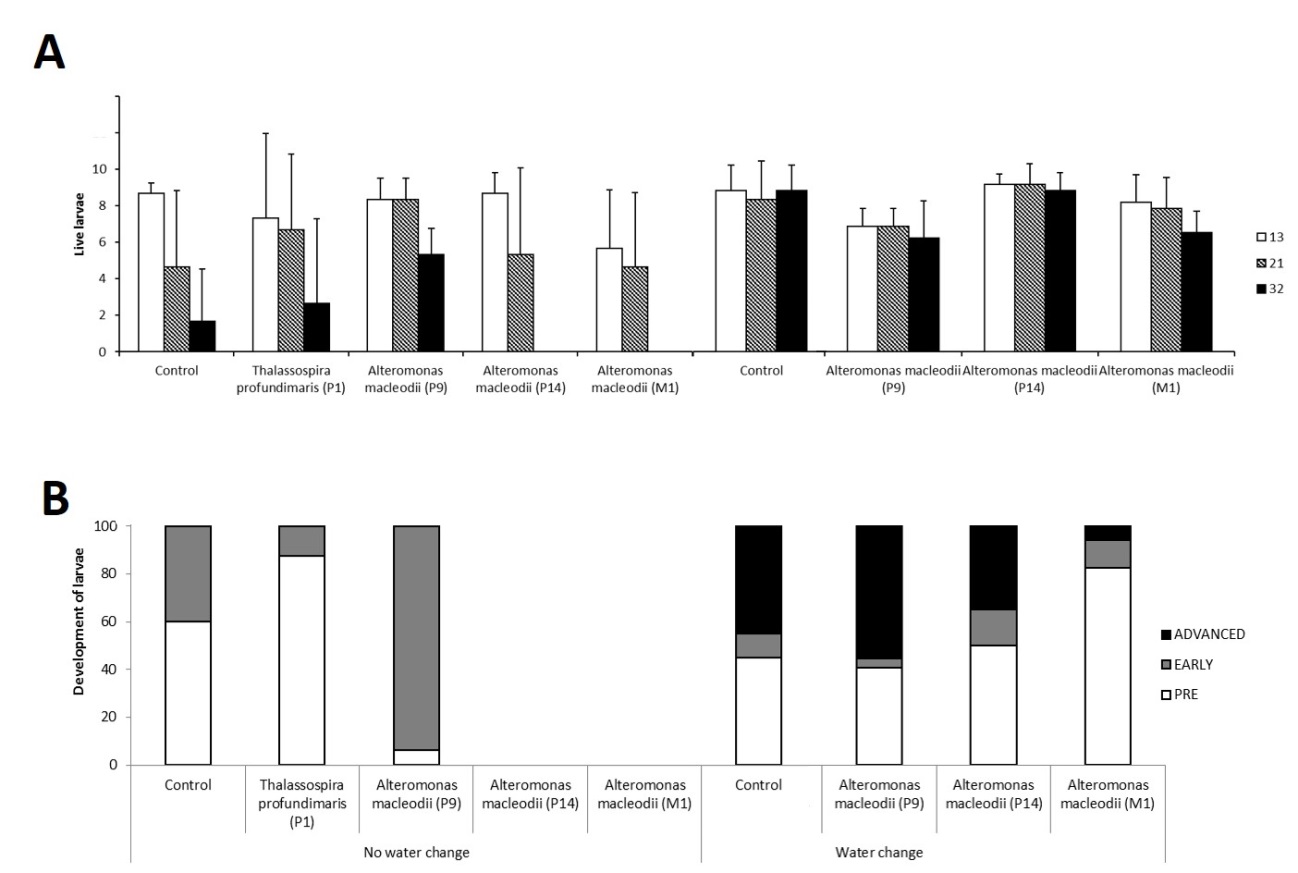


**S6 Fig. Influence of four native bacteria on the survival and development of planula larvae of the octocoral *Rhytisma fulvum fulvum* in cultures with and without water exchange.** Survival (A) and development stage after 32 days (B) with or without water change is shown. The experiment was carried out with planulae of the 2015 batch in 0.22 µm FSW and bacteria were added at a final concentration of 10^3^ CFU mL^-1^.Bacteria were added with every water change. As expected, survival was low in the cultures maintained without water exchange after 32 days. On the contrary, high survival rates were obtained in the control cultures when the water was exchanged, even though the renewal water was free of bacteria (0.22 µm FSW). As in previous experiments, *Alteromonas macleodii* P9 increased survival and development in the planulae cultures maintained without water exchange. When water was changed every 24 hours, none of the *A. macleodii* strains led to an improved survival or development in comparison to the control cultures.
