# Supplementary material for: The effect of bacteria on planula-larvae settlement and metamorphosis in the octocoral Rhytisma fulvum fulvum
Source: PLoS One. 2019 Sep 30;14(9):e0223214. doi: 10.1371/journal.pone.0223214 (PMC6768449; doi:10.1371/journal.pone.0223214)
Supplement: S1 Table — (DOCX) [file pone.0223214.s007.docx]

**S2 Table – Identification of native bacteria isolated from superficial seawater in the Red Sea coral reef seawater tested on the planula larvae cultures of *Rhytisma fulvum fulvum*.**

| **Group** | **Strain** | **Blast closest strain*** | **% ID** | **Family** |
| --- | --- | --- | --- | --- |
| Alpha-Proteobacteria | 86 | Chrobactrum anthropi | 99.11 | Brucellaceae |
|  | 1 | Ruegeria mobilis | 99.33 | Rhodobacteraceae (Roseobacter clade) |
|  | Q5 | Mameliella atlantica | 99.73 | Rhodobacteraceae (Roseobacter clade) |
|  | P1 | Thalassospira profundimaris | 99.11 | Rhodospirillaceae |
| Gamma-Proteobacteria | 118 | Alcanivorax xenomutans | 99.72 | Alcanivoracaceae |
|  | 128 | Alcanivorax xenomutans | 99.64 | Alcanivoracaceae |
|  | A1 | Marinobacter litoralis | 99.71 | Alteromonadaceae |
|  | M1 | Alteromonas macleodii | 98.48 | Alteromonadaceae |
|  | P9 | Alteromonas macleodii | 99.71 | Alteromonadaceae |
|  | P14 | Alteromonas macleodii | 99.71 | Alteromonadaceae |
|  | 22 | Vibrio nereis | 98.91 | Vibrionaceae |
|  | 37 | Vibrio nereis | 98.34 | Vibrionaceae |
|  | 82 | Vibrio nereis | 98.84 | Vibrionaceae |
|  | 52 | Vibrio tubiashii | 99.09 | Vibrionaceae |
|  | 99 | Vibrio variabilis | 98.18 | Vibrionaceae |
| Actinobacteria | A2 | Kokuria rosea | 99.64 | Micrococcaceae |
| Firmicutes | 23 | Planomicrobium chinense | 97.54 | Planococcaceae |

*Closest types species using BLAST search of the EzBioCloud nucleotide sequence database (https://www.ezbiocloud.net/).
